# Correlation Between Retinal Microvascular Abnormalities and Total Magnetic Resonance Imaging Burden of Cerebral Small Vessel Disease in Patients With Type 2 Diabetes

**DOI:** 10.3389/fnins.2021.727998

**Published:** 2021-12-14

**Authors:** Ying Zhang, Zhixiang Zhang, Min Zhang, Yin Cao, Wenwei Yun

**Affiliations:** Department of Neurology, Changzhou Second People’s Hospital Affiliated to Nanjing Medical University, Changzhou, China

**Keywords:** cerebral small vessel disease, retinal microvascular, diabetic retinopathy, type 2 diabetes, retinal photography

## Abstract

**Background and Purpose:** Diabetic retinopathy (DR) is one of the common microvascular complications in diabetes. The total magnetic resonance imaging (MRI) burden of cerebral small vessel disease (CSVD) tends to be increased in diabetic patients and is a marker of microvascular disease; however, the relationship between DR and CSVD is unclear. This study aimed to explore the relationship between retinal microvascular abnormalities and the total MRI burden of CSVD in patients with type 2 diabetes.

**Methods:** Data were collected from patients with type 2 diabetes who were hospitalized between December 2019 and November 2020 in Changzhou Second People’s Hospital affiliated to Nanjing Medical University. All patients underwent retinal photography and cerebral MRI. The central retinal artery equivalent (CRAE), the central retinal venous equivalent (CRVE), and arteriole-to-venule ratio (AVR) were calculated using Image J software to determine the retinal vascular calibers for each patient. The total MRI burden score for CSVD was determined, and the relationship between retinal microvascular abnormalities and the total MRI burden of CSVD was analyzed.

**Results:** Of the 151 diabetic patients included in the study, 84 (55.6%) had no diabetic retinopathy (NDR), 27 (17.9%) had mild DR, and 40 (26.5%) had moderate, or severe non-proliferative DR (grouped together for this study as “more than mild DR”). In patients with more than mild DR, the proportion of moderate to severe burden of CSVD was 75%, which was higher than in patients with mild DR (48.1%) or NDR (26.2%). Patients with moderate to severe burden of CSVD were more likely than those with mild burden of CSVD to have narrowed retinal arterioles (105.24 ± 8.42 μm vs. 109.45 ± 7.93 μm), widened retinal venules (201.67 ± 16.25 μm vs. 193.95 ± 13.54 μm), and lower arteriole-to-venule ratio (0.52 ± 0.05 vs. 0.57 ± 0.04) (*P* < 0.05 for all). The degree of DR (*r* = 0.465, *P* < 0.001) and CRVE (*r* = 0.366, *P* < 0.001) were positively correlated with the total MRI burden of CSVD. Multivariate logistic regression analysis indicated that, after adjustments were made for age, smoking, alcohol consumption, hypertension, and other factors, more than mild DR (OR, 4.383; *P* = 0.028), CRAE (OR, 0.490; *P* = 0.031), and CRVE (OR, 1.475; *P* = 0.041) were independently associated with moderate to severe burden of CSVD.

**Conclusion:** Retinal microvascular abnormalities in patients with type 2 diabetes are associated with the presence of cerebral small vessel lesions. The degree of DR and retinal vessel changes can be used as predictors of intracranial microcirculation lesions.

## Introduction

Type 2 diabetes is a strong risk factor for the development of atherosclerosis. Research has shown that type 2 diabetes is associated with a 2.5-fold increased risk of ischemic stroke, a 1.5-fold increased risk of hemorrhagic stroke, and a 1.5-fold increased risk of dementia compared to the risk in the general population (van Sloten et al., 2020). Type 2 diabetes is also a major risk factor for microvascular dysfunction, including dysfunction in the retinal microvascular system. Specifically, the effect of diabetes is reflected not only in subtle abnormalities of retinal vessels but also in the occurrence of diabetic retinopathy (DR) (Stehouwer, 2018; van Sloten et al., 2020). DR is the most common diabetes-related microvascular complication, with a global prevalence of 34.6% (Shahulhameed et al., 2020).

Research has shown that type 2 diabetes may also be associated with an increasing occurrence of cerebral small vessel disease (CSVD) over time (Qiu et al., 2018; van Agtmaal et al., 2018). CSVD is a slowly progressing disease with non-specific symptoms. Magnetic resonance imaging (MRI) features of CSVD can include white matter hyperintensities (WMHs), lacunes, enlarged perivascular spaces (PVS), and cerebral microbleeds (CMBs), all of which may be a manifestation of cerebral microvascular dysfunction. In recent years, numerous studies have demonstrated a significant correlation between CSVD and retinal vascular changes (McGrory et al., 2019; Shu et al., 2020). Sanahuja et al. (2016) also found that the presence of DR is associated with more severe CSVD. In terms of anatomical features, the retinal arterioles, and venules, measuring 100–300 μm in diameter, share similar features with cerebral small blood vessels. The retinal microvasculature is therefore thought to be a “window” to reflect the condition of the cerebral microvasculature (London et al., 2013).

Given the simultaneous occurrence and joint effects of MRI markers of CSVD, the total MRI burden of CSVD could be used to comprehensively evaluate the cumulative effect of various types of CSVD, with total MRI burden of CSVD potentially offering a better overall assessment of the severity and clinical impact of CSVD (Lau et al., 2017). However, previous studies assessing these potential links usually focused on patients who had experienced stroke; patients with type 2 diabetes have rarely been directly studied. In addition, in previous research assessing the relationship between type 2 diabetes and retinal microvascular abnormalities, the fundus has usually been evaluated via either qualitative assessment of retinopathy signs or quantitative assessment of retinal vessel calibers. In this study, we enrolled only patients with type 2 diabetes and we used retinal photography to assess retinopathy and retinal vascular calibers, thus combining qualitative assessment with quantitative assessment to more fully reflect the abnormalities of the retinal microvasculature. With this study design, we aimed to investigate the correlation between retinal microvascular abnormalities and the total MRI burden of CSVD in patients with type 2 diabetes.

## Materials and Methods

### Study Design

We collected and analyzed data from patients with type 2 diabetes who were hospitalized in the Department of Endocrinology at Changzhou Second People’s Hospital from December 2019 to November 2020. This cross-sectional observational study was approved by the Ethics Committee of Changzhou Second People’s Hospital (2017KY015-01). Informed consent was obtained from patients (or from their family members, if patients were unable to sign the consent form because of illiteracy).

### Patient Selection

A total of 170 patients with type 2 diabetes were screened for our study. These patients did not have dementia and had not experienced stroke. Patients were included if they (1) were aged ≥ 18 years old; (2) met the diagnostic criteria for type 2 diabetes mellitus (Hemmingsen et al., 2017); (3) had cerebral MR images demonstrating any markers of CSVD; and (4) provided informed consent. Patients were excluded if they (1) were unable to complete the cerebral MRI examination or fundus photography; (2) demonstrated evidence of acute cerebral infarcts on cerebral MRI, even if asymptomatic; (3) had any brain disease affecting fundus vessels (e.g., intracranial tumors, arteriovenous malformations, venous sinus thrombosis); (4) had known eye disease or disease that affected the retinal vessel structure or hindered observation of the fundus (e.g., age-related maculopathy, central serous chorioretinopathy, cataract, retinal pigment epithelial detachment) or had undergone previous ophthalmological treatment (e.g., laser photocoagulation, intravitreal injection); (5) had type 1 diabetes or other types of diabetes; (6) had severe organic or metabolic disease; and (7) had unclear fundus images or incomplete cerebral MRI sequences. Of the 170 patients screened for the study, 8 had unclear fundus images, 5 had cataracts or other fundus diseases, 3 had incomplete MRI sequences, and 3 had asymptomatic acute cerebral infarction. These patients were excluded from the analysis, leaving a total of 151 patients enrolled in the study.

### Data Collection

We collected information about patient age, sex, vascular risk factors (including BMI, duration of diabetes, hypertension, smoking, alcohol consumption, previous stroke, coronary heart disease, systolic, and diastolic blood pressure), and laboratory tests such as fasting glucose, glycated hemoglobin (HbA_1_c), total cholesterol, triglyceride, low-density lipoprotein cholesterol, high-density lipoprotein cholesterol, serum creatinine, and urinary microalbumin/creatinine ratio. We also performed carotid ultrasound at baseline for each enrolled participant.

### Analysis of Retinal Microvascular Abnormalities

#### Retinal Vascular Assessment

**FIGURE 1:**
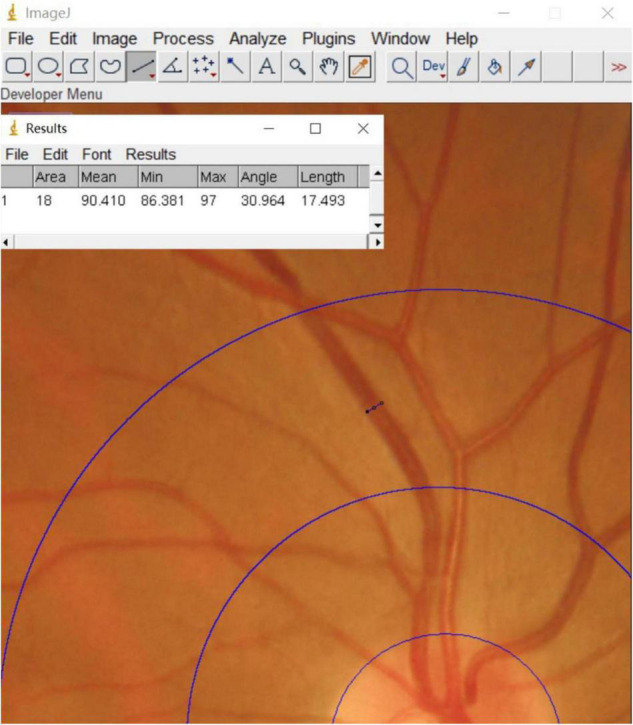
A screenshot of the Image J interface.

A Topcon (TRC-NW400) non-mydriatic fundus camera was used to perform fundus photographic examination of all enrolled patients. Binocular fundus images were obtained with patients sitting under a slit lamp, and the optic disc was confirmed to be in the center of each image. These fundus images were qualitatively analyzed by 2 experienced ophthalmologists. In addition, the software Image J^1^ was used to measure retinal vascular calibers for each patient (Figure 1). The measurement process was completed by a well-trained ophthalmology graduate student. First, all photographs were projected at the same magnification. Next, a 0.5–1 disc diameter surrounding the optic disc was circled and the calibers of the largest 6 retinal arteries and venules were measured. The edge of each blood vessel wall was selected to measure the diameter of the blood vessel vertically. Using this method, we obtained the diameter of 6 retinal arteries and venules. Calibration of the retinal vascular calibers was performed based on the standard disc diameter (1,850 μm) as a defined unit of measurement. Using this uniform conversion, we obtained the vascular calibers close to the true value. Finally, the revised Parr-Hubbard formula was used to calculate the retinal vascular caliber. We used an iterative procedure of pairing up the largest vessels with the smallest and repeating this process until we reached a single number that summarized as the central retinal artery equivalent (CRAE), the central retinal venous equivalent (CRVE), and the arteriole-to-venule ratio (AVR). The following formulas were used to obtain these values (Yip et al., 2016):


$$C\,R\,A\,E=0.88*{\left(W_{1}^{2}+W_{2}^{2}\right)}^{1/2}$$

$$C\,R\,V\,E=0.95*{\left(W_{1}^{2}+W_{2}^{2}\right)}^{1/2}$$

AVR:C⁢R⁢A⁢E/C⁢R⁢V⁢E

#### Diabetic Retinopathy Definition and Classification

**FIGURE 2:**
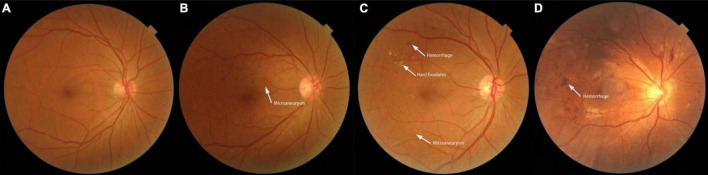
Fundus photographs showing features of a normal fundus and features of the various grades of diabetic retinopathy. **(A)** Photograph of a normal fundus. **(B)** Fundus image showing mild non-proliferative diabetic retinopathy with microaneurysms. **(C)** Fundus image showing moderate non-proliferative diabetic retinopathy with hemorrhages, hard exudates, and microaneurysms. **(D)** Fundus image showing severe non-proliferative diabetic retinopathy.

DR grades (Figure 2) were determined by an experienced ophthalmologist who assessed the cases online, and these grades were reviewed by another experienced ophthalmologist at our hospital; both were blinded to all clinical data and other measurements. Based on an international consensus on clinical DR (Solomon et al., 2017), the grades of DR were defined as follows: 1 = non-DR (NDR), diabetic patients without DR; 2 = mild non-proliferative DR, indicated by microaneurysms only; 3 = moderate non-proliferative DR, indicated by more than just microaneurysms but less than severe non-proliferative DR; 4 = severe non-proliferative DR, indicated by > 20 intraretinal hemorrhages in each of 4 quadrants, definite venous beading in 2 or more quadrants, prominent intraretinal microvascular abnormalities in 1 or more quadrants, or no signs of proliferative retinopathy; and 5 = proliferative DR, indicated by neovascularization and/or vitreous/preretinal hemorrhage. There were no cases of PDR in our study population. For this analysis, DR grades 3 and 4 were combined into a group referred to as “more than mild DR.”

### Analysis of the Total Magnetic Resonance Imaging Burden of Cerebral Small Vessel Disease

Within 7 days after admission, all enrolled patients underwent 3.0T cerebral MRI examination, including T1-weighted imaging (T1WI), T2-weighted imaging (T2WI), fluid attenuation inversion recovery (FLAIR) imaging, diffusion-weighted imaging (DWI), and susceptibility-weighted imaging (SWI).

**FIGURE 3:**
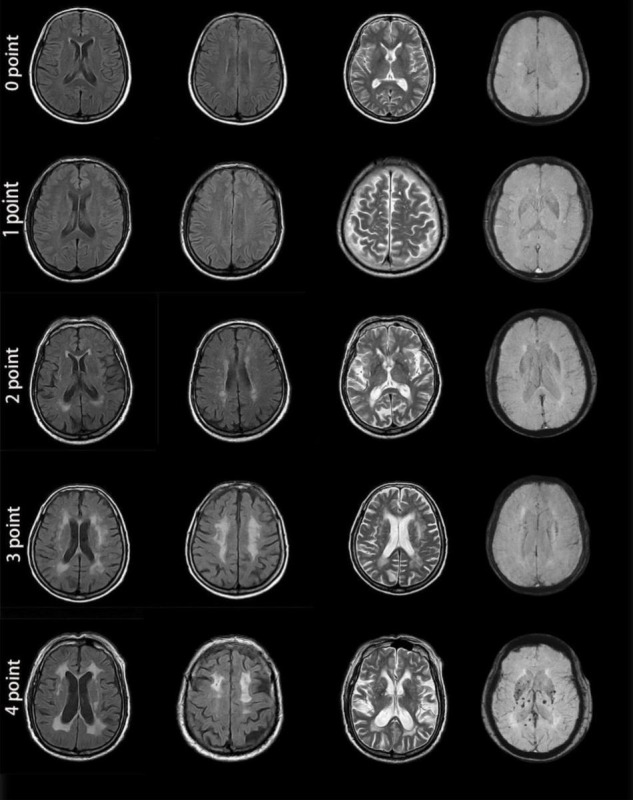
MRI images from patients with different cerebral small vessel disease scores. **Zero points:** This patient was a 46-year-old man with no apparent lesions on MR images. **One point:** This patient was a 56-year-old man. Enlarged perivascular spaces (PVS) (grade 3) could be seen in the brain cortex on T2-weighted imaging. MR images showed no other abnormality. **Two points:** This patient was a 72-year-old woman. MRI demonstrated a lacune in the right basal ganglia, smooth halo-like lesions near the bilateral ventricles (Fazekas 2 for periventricular WMH), confluent white matter hyperintensities (Fazekas 2 for deep WMH) in the deep lobe and enlarged PVS (grade 1) in the bilateral basal ganglia. Susceptibility-weighted imaging showed no abnormality. **Three points:** This patient was a 69-year-old woman. MRI showed irregular white matter lesions in the lateral ventricles extending to the white matter (Fazekas 3 for periventricular WMH), large confluent areas of WMH (Fazekas 3 for deep WMH) in the center of the bilateral semioval and enlarged PVS (grade 3). **Four points:** This patient was a 77-year-old man. There were patchy, irregular white matter lesions extending to the white matter in the lateral ventricle (Fazekas 3 for periventricular WMH), fused WMH (Fazekas 3 for deep WMH), and 2 lacunes in the parietal lobe, enlarged PVS (grade 2) in the bilateral basal ganglia, and cerebral microbleeds at the level of the bilateral ventricles. All 4 MRI markers appeared in the images from this patient.

Two experienced neurologists who were blinded to the clinical information and retinal photography findings independently evaluated all images based on an international consensus (Wardlaw et al., 2013). The total MRI burden scores of CSVD were calculated ranging from 0 to 4 (Figure 3) by combining 4 individual CSVD markers, with 1 point allocated to each of the markers. The specific criteria for the markers were as follows:

(1) WMHs: defined as periventricular or deep brain lesions of varying sizes, hyperintense on T2WI or FLAIR imaging, and isointense or hypointense on T1WI with abnormal white matter signals. The severity of WMHs was assessed using the Fazekas scale. A score of 3 points for hyperintensities in periventricular white matter or ≥ 2 points for hyperintensities in deep white matter was counted as 1 point.
(2) Lacunes: defined as round or oval cerebrospinal fluid-like signals on T1WI and T2WI, with a surrounding rim of hyperintensities and central cerebrospinal fluid-like hypointensities on FLAIR imaging, with a diameter of 3–15 mm, distributed under the cortex. The presence of ≥ 1 lacune was counted as 1 point.
(3) CMBs: defined as round or oval signal loss lesions on SWI, with clear boundaries, mostly 2–5 mm in diameter, located in the cortico-subcortical junction and deep in the cerebral hemispheres. The presence of ≥ 1 CMB was counted as 1 point.
(4) PVS: defined as round, oval, or linear lesions that pass-through of gray or white matter, hypointense on T1WI and FLAIR imaging, and hyperintense on T2WI, with a diameter of < 3 mm. A visual quantization method was used to count the number of lesions in the basal ganglia and semioval center. A PVS of level ≥ 2 was counted as 1 point (Huijts et al., 2013; Lau et al., 2017).

The total MRI burden of CSVD scores was then categorized into 2 groups based on the simple CSVD score: mild burden (0–1 points) or moderate to severe burden (2–4 points).

### Statistical Analysis

All statistical analyses were performed with the Windows SPSS software package (Version 26.0, IBM Corporation, Armonk, NY, United States). Continuous variables with normal distribution were presented as mean ± SD, whereas continuous variables with skewed distribution were summarized as medians and interquartile ranges. Categorical variables were expressed as numbers and percentages. Differences between groups were tested in univariate analyses using independent sample *t*-test, Kruskal-Wallis *H*-test, Mann-Whitney *U*-test, chi-square test, or Fisher’s exact test as appropriate. LSD tests and Bonferroni corrections were used in *post hoc* analyses. Variables that demonstrated a degree of significance of *P* < 0.1 in univariate analysis were entered into a multivariate binary logistic regression model to analyze the relationship between retinal microvascular abnormalities and the total MRI burden of CSVD. The correlation between the total MRI burden of CSVD and DR degree and retinal vascular calibers was tested using the Spearman rank method. Significance was defined as *P <* 0.05.

## Results

### Baseline Characteristics of Diabetic Retinopathy

Among the 151 patients included in the study, the average age was 63.9 ± 8.6 years. There were 84 (55.6%) patients with NDR, 27 (17.9%) patients with mild DR, and 40 (26.5%) patients with moderate, severe, or proliferative DR (more than mild DR). Patients with more than mild DR were older and had a longer duration of diabetes, higher systolic blood pressure, and greater urinary microalbumin excretion than those with NDR (Table 1). No significant difference was observed among groups in sex distribution, medication use, or other baseline data.

**TABLE 1:** Clinical characteristics of the study population according to DR status (NDR, mild DR, or more than mild DR).

Variables	NDR (*n* = 84)	Mild DR (*n* = 27)	More than mild DR (*n* = 40)	*P*-value
Age, y; mean ± SD	62.3 ± 8.6	65.5 ± 8.0	66.3 ± 8.2*	0.030
Male, n (%)	44 (52.4)	14 (51.9)	16 (40.0)	0.413
**Vascular risk factors at baseline**				
Smoking, n (%)	22 (23.8)	11 (40.7)	9 (22.5)	0.178
Alcohol consumption, n (%)	14 (16.7)	6 (22.2)	9 (22.5)	0.675
Hypertension, n (%)	51 (60.7)	18 (66.7)	27 (67.5)	0.714
Duration of diabetes, years, median (IQR)	10 (4, 15)	12 (10, 20)	19.5 (10, 21)*	<0.001
Previous stroke, n (%)	14 (16.7)	5 (18.5)	9 (22.5)	0.737
Coronary heart disease, n (%)	6 (7.1)	2 (7.4)	0 (0)	0.218
Carotid artery plaques, n (%)	51 (60.7)	19 (70.4)	29 (72.5)	0.367
Systolic BP, mmHg, mean ± SD	135 ± 18	129 ± 17	143 ± 21*^§^	0.009
Diastolic BP, mmHg, mean ± SD	78 ± 10	75 ± 12	78 ± 11	0.314
BMI, kg/m^2^, mean ± SD	24.16 ± 3.57	23.58 ± 2.92	23.88 ± 3.11	0.715
**Laboratory tests**				
Fasting glucose, mmol//L, median (IQR)	8.38 (6.62, 11.30)	8.06 (6.29, 12.09)	8.79 (6.89, 11.70)	0.774
HbA_1_c, %, median (IQR)	8.40 (7.73, 10.50)	8.40 (7.30, 10.50)	8.45 (7.53, 9.95)	0.880
Total cholesterol, mmol//L, mean ± SD	4.29 ± 1.02	4.60 ± 1.59	4.46 ± 1.34	0.492
Triglycerides, mmol//L, mean ± SD	1.86 ± 1.17	1.81 ± 1.41	1.70 ± 1.02	0.771
LDL-C, mmol//L, mean ± SD	2.42 ± 0.71	2.56 ± 0.90	2.55 ± 0.87	0.605
HDL-C, mmol//L, mean ± SD	1.04 ± 0.29	1.14 ± 0.37	1.10 ± 0.30	0.268
Serum creatinine, μmol//L, mean ± SD	63.26 ± 19.10	67.76 ± 28.84	73.60 ± 34.14	0.112
Urinary microalbumin/creatinine ratio, mg/g, median (IQR)	11.80 (7.15, 28.73)	27.00 (8.40, 58.40)*	20.80 (9.33, 163.65)*	0.012
**Medication use**				
Insulin treatment, n (%)	25 (29.8)	6 (22.2)	15 (37.5)	0.402
Hypoglycemic agents, n (%)	48 (57.1)	14 (51.9)	20 (50.0)	0.727
Antihypertensive medication, n (%)	30 (35.7)	11 (40.7)	17 (42.5)	0.740
Antiplatelet medication, n (%)	15 (17.9)	8 (29.6)	6 (15.0)	0.294
Lipid-modifying medication, n (%)	28 (33.3)	11 (40.7)	10 (25.0)	0.389
**Retinal vessel changes**				
CRAE, μm, mean ± SD meanSD	109.15 ± 8.96	106.37 ± 6.70	105.32 ± 7.62*	0.039
CRVE, μm, mean ± SD	191.58 ± 16.47	200.34 ± 9.63*	207.15 ± 8.59*	<0.001
AVR, mean ± SD	0.57 ± 0.04	0.53 ± 0.04*	0.51 ± 0.04*^§^	<0.001
**Total MRI burden score of CSVD**				<0.001
Mild burden, n (%)	62 (73.8)	14 (51.9)	10 (25.0)*	
Moderate to severe burden, n (%)	22 (26.2)	13 (48.1)	30 (75.0)*	

*DR, diabetic retinopathy; NDR, no diabetic retinopathy; SD, standard deviation; IQR, interquartile range; LDL, low-density lipoprotein; HDL, high-density lipoprotein; CRAE, Central Retinal Arterial Equivalent; CRVE, Central Retinal Venous Equivalent; AVR, arteriole-to-venule ratio; CSVD, cerebral small vessel disease.*

*Indicates that the difference was statistically significant compared with the NDR group.

*^§^Indicates that the difference was statistically significant compared with the mild DR group.*

**FIGURE 4:**
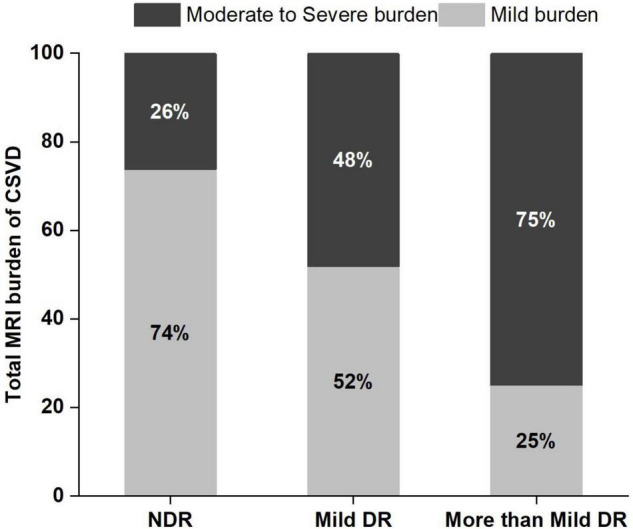
Frequency and severity of cerebral small vessel disease (CSVD) in the 3 study groups. DR, diabetic retinopathy; NDR, no diabetic retinopathy.

The proportion of moderate to severe burden of CSVD in the more than mild DR group was 75%, which was higher than that of the mild DR group (48.1%) and the NDR group (26.2%) (Figure 4). There was a significant tendency toward retinal arteriolar narrowing and venular widening with increasing degree of DR. The AVR values in patients with mild DR (0.53 ± 0.04) and in those with more than mild DR (0.51 ± 0.04) were smaller than in patients with NDR (0.57 ± 0.04). These significant associations remained significant after LSD tests or Bonferroni corrections (Table 1).

### Association of Retinal Microvascular Abnormalities With Total Magnetic Resonance Imaging Burden of Cerebral Small Vessel Disease

A total of 86 patients demonstrated mild burden of CSVD, and 65 patients demonstrated moderate to severe burden of CSVD. Patients with moderate to severe CSVD burden were more likely than those with mild CSVD burden to have narrowed retinal arterioles (105.24 ± 8.42 μm vs. 109.45 ± 7.93 μm), widened retinal venules (201.67 ± 16.25 μm vs. 193.95 ± 13.54 μm), and lower AVR (0.52 ± 0.05 vs. 0.57 ± 0.04) (*P* < 0.05 for all). Patients with moderate to severe burden of CSVD also demonstrated a higher proportion of more than mild DR (46.2%) than those with mild burden of CSVD (11.6%) (Table 2).

**TABLE 2:** Clinical characteristics of the study population based on the total MRI burden of CSVD.

Variables	Mild burden (*n* = 86)	Moderate to severe burden (*n* = 65)	*P*-value
Age, y; mean ± SD	61.3 ± 8.7	67.4 ± 7.2	<0.001
Male, n (%)	43 (50.0)	31 (47.7)	0.779
**Vascular risk factors at baseline**			
Smoking, n (%)	17 (19.8)	23 (35.4)	0.031
Alcohol consumption, n (%)	11 (12.8)	18 (27.7)	0.021
Hypertension, n (%)	44 (51.2)	52 (80.0)	<0.001
Duration of diabetes, years, median (IQR)	10 (6, 17)	12 (6.5, 20.5)	0.113
Previous stroke, n (%)	7 (8.1)	21 (32.3)	<0.001
Coronary heart disease, n (%)	4 (4.7)	4 (6.2)	0.726
Carotid artery plaques, n (%)	47 (54.7)	52 (80.0)	0.001
Systolic BP, mmHg, mean ± SD	133 ± 18	141 ± 21	0.008
Diastolic BP, mmHg, mean ± SD	77 ± 11	78 ± 10	0.741
BMI, kg/m^2^, mean ± SD	24.05 ± 3.12	23.90 ± 3.61	0.781
**Laboratory test**			
Fasting glucose, mmol//L, median (IQR)	7.78 (6.47, 9.89)	9.37 (7.47, 12.77)	0.005
HbA_1_c, %, median (IQR)	8.10 (7.0, 9.68)	9.40 (8.05, 10.90)	<0.001
Total cholesterol, mmol//L, mean ± SD	4.47 ± 1.17	4.28 ± 1.30	0.350
Triglycerides, mmol//L, mean ± SD	1.83 ± 1.12	1.79 ± 1.25	0.846
LDL-C, mmol//L, mean ± SD	2.48 ± 0.75	2.36 ± 0.83	0.475
HDL-C, mmol//L, mean ± SD	1.11 ± 0.32	1.02 ± 0.28	0.086
Serum creatinine, μmol//L, mean ± SD	60.95 ± 19.46	74.54 ± 30.93	0.001
Urinary microalbumin/creatinine ratio, mg/g, median (IQR)	11.80 (6.60, 27.58)	20.50 (9.10, 97.95)	<0.001
**Retinal microvascular abnormalities**			
DR			<0.001
NDR, n (%)	62 (72.1)	22 (33.8)	
Mild DR, n (%)	14 (16.3)	13 (20.0)	
More than mild DR, n (%)	10 (11.6)	30 (46.2)	
CRAE, μm, mean ± SD	109.45 ± 7.93	105.24 ± 8.42	0.002
CRVE, μm, mean ± SD	193.95 ± 13.54	201.67 ± 16.25	0.002
AVR, mean ± SD	0.57 ± 0.04	0.52 ± 0.05	<0.001

*DR, diabetic retinopathy; NDR, no diabetic retinopathy; SD, standard deviation; IQR, interquartile range; LDL, low-density lipoprotein; HDL, high-density lipoprotein; CRAE, Central Retinal Arterial Equivalent; CRVE, Central Retinal Venous Equivalent; AVR, arteriole-to-venule ratio; CSVD, cerebral small vessel disease.*

Spearman rank correlation analysis demonstrated that the degree of DR (*r* = 0.465, *P* < 0.001) and CRVE (*r* = 0.366, *P* < 0.001) were positively correlated with the total MRI burden of CSVD, whereas CRAE (*r* = –0.306, *P* < 0.001) was negatively correlated with the total MRI burden of CSVD.

### Multivariate Logistic Regression Analysis of Total Magnetic Resonance Imaging Burden of Cerebral Small Vessel Disease

Multivariate logistic regression analysis was performed to further evaluate the association between the total MRI burden of CSVD and retinal microvascular abnormalities. After adjustments were made for confounding factors such as age, smoking, alcohol consumption, hypertension, and stroke, more than mild DR (OR, 4.383; 95% CI, 1.179–17.202; *P* = 0.028), CRAE (OR, 0.490; 95% CI, 0.256–0.936; *P* = 0.031), and CRVE (OR, 1.475; 95% CI, 1.016–2.143; *P* = 0.041) were found to be independently associated with moderate to severe burden of CSVD (Table 3).

**TABLE 3:** Multivariate logistic regression analysis of the total MRI burden of CSVD.

Variable	OR	95% CI	*P*-value
Age	1.100	1.024∼1.181	0.009
Hypertension	3.531	1.118∼11.148	0.031
HbA_1_c	1.601	1.117∼2.294	0.010
More than mild DR	4.383	1.179∼17.202	0.028
CRAE	0.490	0.256∼0.936	0.031
CRVE	1.475	1.016∼2.143	0.041

*OR, odds ratio; CI, confident interval; DR, diabetic retinopathy; CRAE, Central Retinal Arterial Equivalent; CRVE, Central Retinal Venous Equivalent; CSVD, cerebral small vessel disease.*

## Discussion

This study found that DR was correlated with the total MRI burden of CSVD in patients with type 2 diabetes. More specifically, the degree of DR was associated with more severe CSVD. Further, CRAE, CRVE, and the presence of more than mild DR were independently associated with increased burden of CSVD.

DR has a high incidence in patients with diabetes and is one of the most common microvascular complications in this population. Additionally, as a slowly progressive neuromicrovascular disorder, diabetes is associated with an increased risk of the occurrence of CSVD (van Sloten et al., 2020). Research has shown that retinal microvascular abnormalities can reflect changes in small cerebral arteries caused by vascular risk factors such as diabetes and hypertension (Yip et al., 2016). Previous studies have mostly focused on the relationship between retinal microvascular abnormalities and individual CSVD markers. Mutlu et al. (2016) found that the widening of retinal venules and arteriole stenosis were related to the volume of white matter lesions and that changes in retinal vessels calibers may have predated these lesions. Dumitrascu et al. (2018) found that changes in arteriovenous nicking, focal arteriolar narrowing, and retinal vascular curvature were more common in patients with CSVD than in those without CSVD. The degree of retinal vein dilation and focal arteriolar narrowing was related to the presence of lacunes. In our study, we used a scoring system that included WMHs, lacunes, EPVS, and cerebral microhemorrhages in the assessment of CSVD. It is a relatively new scoring system in recent years that can comprehensively evaluate the combined effect of CSVD lesions. The effectiveness and applicability of this scoring system for CSVD have been demonstrated in a growing number of studies (Lau et al., 2017; Yang et al., 2019; Shu et al., 2020).

Previous research has shown that retinal microvascular dysfunction is related to diabetes mellitus; this dysfunction includes not only DR but also subtle abnormalities in the structure and function of retinal microvascular vessels, such as retinal venule dilation or arteriole reduction, and increased fractal dimension (Stehouwer, 2018; van Sloten et al., 2020). In our study, differences in retinal blood vessel calibers were observed among patients with different severities of DR. With the aggravation of DR, CRVE tended to increase, whereas CRAE and AVR tended to decrease. These differences were statistically significant, suggesting that widened retinal venules and narrowed retinal arterioles may be related to the progression of DR. Diabetes is associated with a number of microvascular and macrovascular complications that affect the retina and the brain in parallel, so both retinal blood vessels and cerebrovascular vessels are susceptible to this disease (Pearce et al., 2019). Previous research has shown that retinopathy and retinal microvascular abnormalities are associated with the presence and progression of CSVD in patients with type 2 diabetes (Cheung et al., 2015), some of the findings confirmed by our study. In our study, we found that DR was related to the total MRI burden of CSVD, and the more severe the DR was, the heavier the total MRI burden was.

Hyperglycemia is associated with systemic endothelial dysfunction of the microcirculation, which can cause cerebral hypoperfusion, leading to chronic cerebral ischemia (Sörensen et al., 2016). The changes in cerebral microcirculation in diabetic patients are related to the increased permeability of the blood-brain barrier and changes in cerebral blood flow regulation. The blood-brain barrier is susceptible to oxidative stress, which may be caused by the increased production of reactive oxygen species associated with hyperglycemia and limited antioxidant defenses in the brain. This disruption of the blood-brain barrier in turn leads to vessel wall thickening and disorders of the cerebral microcirculation, resulting in structural brain abnormalities (Bogush et al., 2017; Rhea and Banks, 2019). In our study, we found that patients with narrower retinal arterioles and wider retinal venules show a heavier total MRI burden of CSVD. After adjusting for confounding factors such as age, smoking, alcohol consumption, hypertension, and stroke, we found that CRAE, CRVE, and the presence of more than mild DR were independently associated with moderate to severe burden of CSVD.

Patients with diabetes may also be affected by microvascular complications other than DR, such as diabetic nephropathy and diabetic neuropathy. A systematic review reported that DR is consistently associated with other complications of diabetes, with the severity of DR contributing to a higher risk of developing other microvascular complications (Pearce et al., 2019). One study found that nephropathy was the only complication of diabetes independently associated with DR, and the presence of retinopathy increased the likelihood of developing nephropathy by 4.37 times (El-Asrar et al., 2001). In our study, we also found that patients with diabetes who have retinopathy, compared with those without retinopathy, had greater urinary albumin excretion. Fewer studies have evaluated the relationship between retinal vascular changes and diabetic neuropathy. One population-based cross-sectional study found that suboptimal arteriolar caliber and DR were associated with peripheral neuropathy (Ding et al., 2012).

This study also found that age and hypertension were independent risk factors for increased total MRI burden of CSVD, which is consistent with the results of previous studies (Hernandez-Diaz et al., 2019; Shu et al., 2020). Nam et al. (2020) found that in patients with no history of cerebrovascular disease, a high triglyceride-glucose index was associated with a higher total burden of CSVD, suggesting that this index may be a convenient and useful predictor of CSVD. In a large population-based study, van Agtmaal et al. (2018) found that persistent hyperglycemia was associated with abnormalities such as WMHs and lacunes. In our study, HbA1c was found to be an independent risk factor for moderate to severe burden of CSVD, which was consistent with the findings of van Agtmaal et al. (2018). However, we observed no significant correlation between triglycerides or fasting blood glucose and the total MRI burden of CSVD; this lack of association may be related to the use of medications to control diabetes, as well as lipid-regulating agents.

This study had several limitations. First, because this was a cross-sectional study, a direct relationship between retinal microangiopathy and CSVD could not be demonstrated. Second, the study included patients with type 2 diabetes but did not include age-matched prediabetic or non-diabetic controls, making it difficult to extrapolate these results to a wider population. Future studies will be needed to assess the relationship between total MRI burden and retinal microvascular lesions in patients with prediabetes and non-vascular factors. The semiquantitative method used in this study to evaluate retinal blood calibers may be inaccurate. In addition, while some studies have adopted quantitative assessment of WMHs, there is no quantitative disease burden evaluation system that truly targets small blood vessels themselves. Further research is needed to establish a more accurate system for evaluating the total MRI burden of CSVD. In future studies, we plan to use a high-resolution, high-quality imaging segmentation method to examine volumetric data for WMH, lacunes, EPVS, recent subcortical infarcts, microbleeds, and global and regional brain volume, with the goal of identifying the global burden of brain changes. Automated image quantification tools are becoming a crucial part of clinical research and practice; thus, a robust and precise MRI segmentation method capable of identifying multiple imaging features of CSVD is needed. Finally, further research is needed regarding quantifying and intelligentizing imaging and symptomatic diagnosis for CSVD.

## Conclusion

In conclusion, this study found that retinal microvascular abnormalities in diabetic patients are related to the occurrence of CSVD. These retinal microvascular abnormalities can be used to evaluate the severity of CSVD and to predict the occurrence of intracranial microvascular disease.

## Data Availability Statement

The original contributions presented in the study are included in the article/supplementary material, further inquiries can be directed to the corresponding author.

## Ethics Statement

This cross-sectional observational study was approved by the Ethics Committee of Changzhou Second People’s Hospital (2017KY015-01). Informed consent was obtained from patients or their family members. The patients/participants provided their written informed consent to participate in this study.

## Author Contributions

YZ, ZZ, and MZ designed the study. YC, ZZ, and WY reviewed clinical MRIs for radiologic grading. YZ carried out data analysis. YZ and ZZ wrote the manuscript. MZ, YC, and WY made the important data analysis suggestions and manuscript revisions. All authors contributed to the article and approved the submitted version.

## Conflict of Interest

The authors declare that the research was conducted in the absence of any commercial or financial relationships that could be construed as a potential conflict of interest.

## Publisher’s Note

All claims expressed in this article are solely those of the authors and do not necessarily represent those of their affiliated organizations, or those of the publisher, the editors and the reviewers. Any product that may be evaluated in this article, or claim that may be made by its manufacturer, is not guaranteed or endorsed by the publisher.

## Footnotes

1
https://imagej.en.softonic.com/
